# Reconstruction of thermotolerant yeast by one-point mutation identified through whole-genome analyses of adaptively-evolved strains

**DOI:** 10.1038/srep23157

**Published:** 2016-03-17

**Authors:** Atsushi Satomura, Natsuko Miura, Kouichi Kuroda, Mitsuyoshi Ueda

**Affiliations:** 1 Division of Applied Life Sciences, Graduate School of Agriculture, Kyoto University, Sakyo-ku, Kyoto, Japan; 2 Japan Society for the Promotion of Science, Sakyo-ku, Kyoto, Japan

## Abstract

*Saccharomyces cerevisiae* is used as a host strain in bioproduction, because of its rapid growth, ease of genetic manipulation, and high reducing capacity. However, the heat produced during the fermentation processes inhibits the biological activities and growth of the yeast cells. We performed whole-genome sequencing of 19 intermediate strains previously obtained during adaptation experiments under heat stress; 49 mutations were found in the adaptation steps. Phylogenetic tree revealed at least five events in which these strains had acquired mutations in the *CDC25* gene. Reconstructed *CDC25* point mutants based on a parental strain had acquired thermotolerance without any growth defects. These mutations led to the downregulation of the cAMP-dependent protein kinase (PKA) signaling pathway, which controls a variety of processes such as cell-cycle progression and stress tolerance. The one-point mutations in *CDC25* were involved in the global transcriptional regulation through the cAMP/PKA pathway. Additionally, the mutations enabled efficient ethanol fermentation at 39 °C, suggesting that the one-point mutations in *CDC25* may contribute to bioproduction.

In bioproduction, heat produced during fermentation diminishes the cellular growth and fermentation rates of yeasts. Since it is not cost-effective to cool down the fermentation apparatus to maintain the efficiency, thermotolerant yeast strains that are capable of growth at high temperatures are required^1^. S*accharomyces cerevisiae* is a widely used host strain for bioproduction, because of its rapid growth under aerobic and anaerobic conditions, ease of genetic manipulation, and high reducing capacity^2^. Despite these advantages, little is known about the mutations involved in thermotolerance. Genomic mutations that confer the yeast with thermotolerance are required for efficient bioproduction^3^.

Experimental evolution is one of the effective methods to connect genotypes to phenotypes^4^. The outcomes obtained from an experimental evolution potentially facilitate the rational engineering of productive strains for the bioindustry. However, identification of the mutations associated with the phenotype sometimes requires laborious works, especially when evolved strains have acquired a variety of mutations. Although the development of deep-sequence technologies has enabled comprehensive whole-genome analyses of microorganisms^5^, it is still difficult to select candidate mutations involved in the phenotypes from the total of all identified mutations.

Previously, we have performed stepwise breeding under heat stress and obtained a thermotolerant strain^6^. In this breeding, a non-thermotolerant strain, MT8-1, was successively cultured at 32 °C, 34 °C, 36 °C, and 38 °C until the cells adapted to the temperatures. The strain isolated at 38 °C showed thermotolerance and accumulation of trehalose. We preserved intermediate populations that adapted to each of the temperatures ranging from 32 °C to 38 °C. These intermediate strains allowed us to track the dominant strains at each temperature and to analyze the genomic mutations that the adapted strains have acquired. Whole-genome sequencing of these strains helped us identify the mutations involved in global transcriptional regulation and thermotolerance.

In *Saccharomyces cerevisiae*, the cAMP-dependent protein kinase (PKA) signaling pathway has been reported to regulate thermotolerance^7^. The cAMP/PKA signaling pathway is a well conserved pathway, operated by intracellular cAMP as a second messenger^8^. cAMP/PKA pathway controls a variety of processes including cell-cycle progression^9^, life span^10^, diauxic shift^11^, and stress response^7^. Intracellular cAMP level is regulated by adenylate cyclase (Cyr1p), which converts ATP to cAMP^12^. The monomeric G proteins (Ras1p and Ras2p) control the activity of Cyr1p depending on the activity of Cdc25p^13^. Cdc25p is a membrane bound guanine nucleotide exchange factor (GEF) that activates Ras1p and Ras2p by stimulating the release of GDP and the binding of GTP^14^. Lower cAMP level activates the stress responsive transcriptional activators such as Msn2p and Msn4p, resulting in stress tolerance^7^. However, excessive downregulation of the cAMP/PKA pathway leads to a trade-off between thermotolerance and diminished growth rate, because the cell cycle is also under the control of this pathway^15^.

In this study, we analyzed genomic sequences of intermediate strains to track the dominant strains during the stepwise adaptation. Four mutations in the *CDC25* gene were revealed to play a critical role in thermotolerance through downregulation of intracellular cAMP levels. Reconstructed *CDC25* one-point mutants based on the parental strain MT8-1 exactly exhibited thermotolerance without any major growth defects. These *CDC25* mutants were able to produce more ethanol from galactose at 30 °C than MT8-1. In addition, they retained the ethanol fermentation rates from glucose and galactose even at 39 °C, unlike parental strain MT8-1. These mutations in *CDC25* will be beneficial for bioproduction under heat stress conditions.

## Results

### Identification of a key gene for thermotolerance of yeast

**Figure 1:**
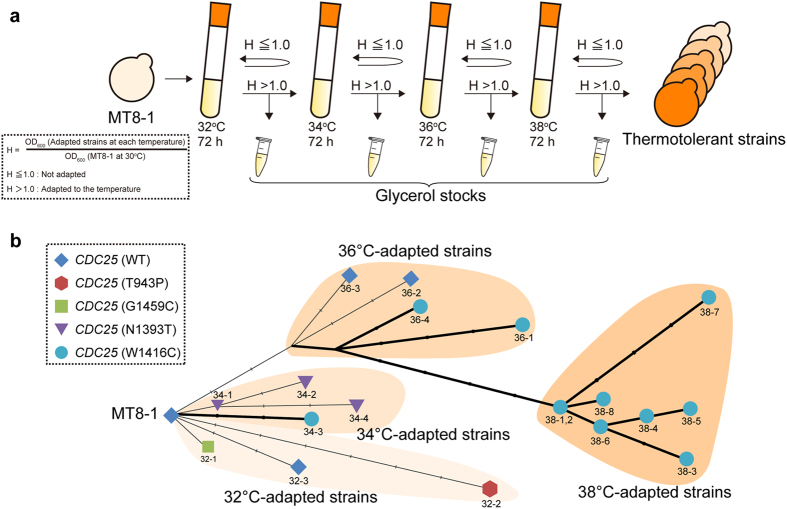
Stepwise breeding of thermotolerant yeast strains. (**a**) Schematic illustration of stepwise breeding. Yeast cells were continuously cultivated at each temperature until the cells had adapted to the temperature (H > 1.0). (**b**)Phylogenetic tree constructed based on the mutational events of the intermediate strains. The small scales indicate each mutational event (base substitutions, insertions, and deletions). The bold lines indicate the cell lines that had acquired a *CDC25* (W1416C) mutation. *CDC25* (W1416C) appeared independently in two populations. The strain names correspond to the names in Supplementary Table S2.

In order to identify mutations that contribute to thermotolerance in *S. cerevisiae*, we sequenced the whole-genome of the parental strain and 19 intermediate strains isolated in the stepwise breeding^6^ (Fig. 1a, DDBJ accession number DRA004175). Forty-nine genomic mutations including SNPs, insertions, and deletions were identified in the intermediate strains by comparing them with the genomic sequence of MT8-1. Inversions and other rearrangements were not observed in our analysis (Supplementary Table S2). The genome analyses revealed that the yeast cells had acquired four different point mutations in the “*CDC25* gene”. We constructed a phylogenetic tree according to each of the mutational events in the intermediate strains to analyze how the *CDC25* mutants had developed in the adaptational steps (Fig. 1b). The phylogenetic tree suggests that there were at least five events (T943P, G1459C, N1393T, and twice in W1416C) in which the bred strains had obtained mutations in the *CDC25* gene. The phylogenetic tree implied that the mutants harboring the *CDC25* (W1416C) mutation appeared independently at 34 °C and 36 °C. It is highly rare that the several strains have acquired the mutations in the same gene locus, since genomic mutations basically occur randomly in 1.2 × 10^7^ base-pair genome of *S. cerevisiae*. Therefore, we considered that the frequently appeared *CDC25* mutations in the stepwise adaptation would play a crucial role in thermotolerance.

**Figure 2:**
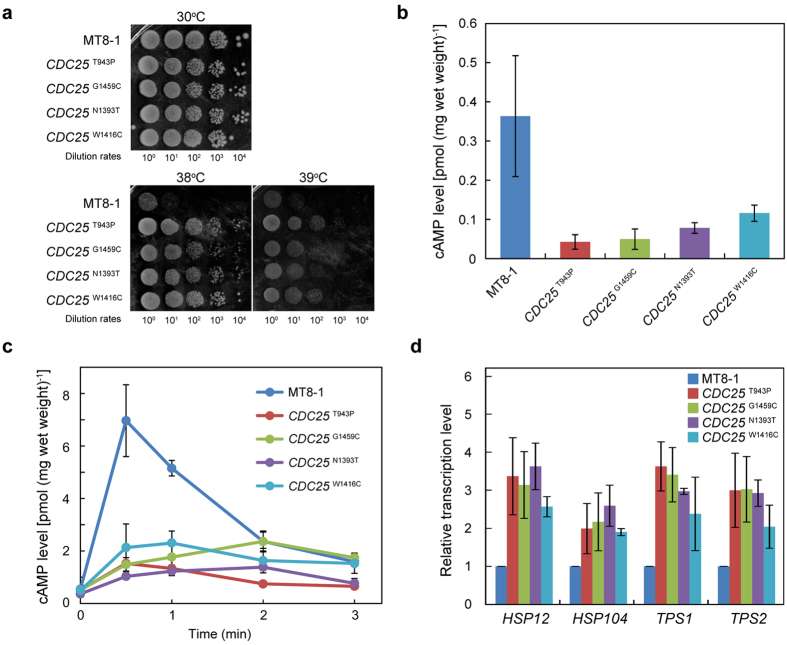
Characteristics of the reconstructed *CDC25* mutants based on MT8-1. (**a**) Spot assay. Cells (OD_600_ = 1.0) were diluted 10-, 10^2^-, 10^3^-, and 10^4^-fold and spotted onto a YPD plate. (**b**) Basal cAMP levels. (**c**) Induced cAMP levels by glucose. Following the addition of glucose, the cAMP levels were measured at each time point. (**d**) Relative transcription levels of *HSP12*, *HSP104*, *TPS1*, and *TPS2* measured by real-time PCR. The error bars show standard error of the mean (SEM) based on three independent measurements.

To examine this hypothesis, we introduced each *CDC25* mutation into the parental strain and reconstructed *CDC25* mutants based on MT8-1 (hereafter referred to as *CDC25*^T943P^, *CDC25*^G1459C^, *CDC25*^N1393T^, and *CDC25*^W1416C^). All the reconstructed *CDC25* mutants grew better at 38 °C and 39 °C in contrast to MT8-1 (Fig. 2a, Supplementary Fig. S1). Thermotolerance of the *CDC25* mutants was enough stable and was maintained during the cellular growth.

Cdc25p, known as guanine nucleotide exchange factor (GEF), indirectly regulates intracellular cAMP levels and thus, the cAMP/PKA signaling pathway. The cAMP/PKA pathway is responsible for inactivation of the Msn2p/Msn4p transcriptional activators that control general stress responses in *S. cerevisiae*. Since lower levels of intracellular cAMP activate Msn2p/Msn4p, we considered that the mutations in Cdc25p decreased the GEF activity and intracellular cAMP levels. In fact, the reconstructed *CDC25* mutants showed lower intracellular cAMP levels than the wild-type parental strain (Fig. 2b). The decreased activity of the *CDC25* mutants was also confirmed by measuring the glucose-responsiveness (Fig. 2c). Addition of glucose to the reconstructed *CDC25* mutants did not trigger a rapid increase in the cAMP levels unlike parental MT8-1, indicating that the mutated Cdc25p is involved in lowering of the intracellular cAMP levels. To further validate the activation of Msn2p and Msn4p in the reconstructed *CDC25* mutants, the transcriptional levels of *HSP12*, *HSP104*, *TPS1*, and *TPS2* were measured (Fig. 2d). These genes are induced by Msn2p/Msn4p through the upstream stress-responsive elements (STREs)^16^. *Hsp12p* is a membrane protein involved in membrane organization under heat stress^17^. *Hsp104p* acts as a molecular chaperone responsible for refolding of denatured and aggregated proteins^18^. *Tps1p* and *Tps2p* are the subunits of trehalose-6-P synthase complex^19^. The reconstructed *CDC25* mutants showed higher transcriptional levels of these genes, suggesting that the enhanced Msn2p and Msn4p activities resulted in thermotolerance of the *CDC25* mutants.

### Transcriptome analysis

**Figure 3:**
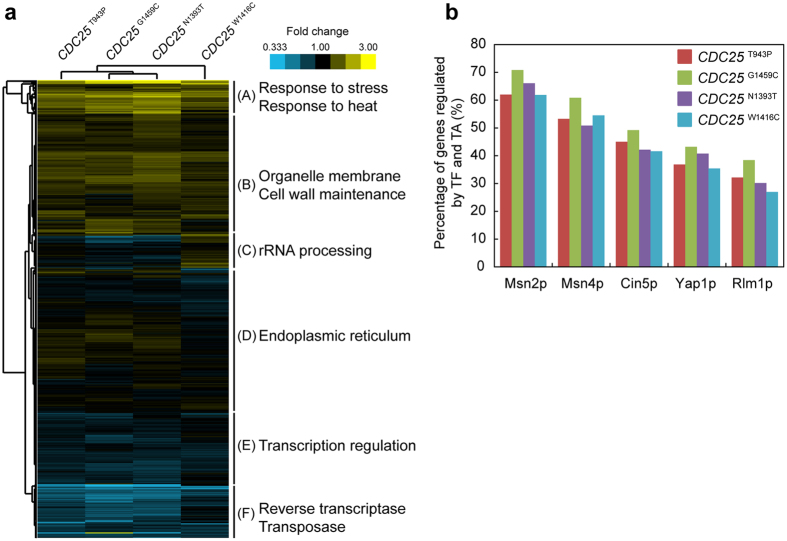
mRNA profiles of the reconstructed *CDC25* mutants in comparison with that of MT8-1. (**a**) Hierarchical clustering analysis. The gene expressions of the reconstructed *CDC25* mutants compared to that of MT8-1 were classified by hierarchical clustering. (**b**) Transcription factor (TF) and transcriptional activator (TA) analyses involved in the expressions of more than 1.5-fold induced genes compared to that in MT8-1. The y-axis indicates the percentage of the genes controlled by each of the transcriptional regulator among the more than 1.5-fold induced genes.

Transcriptome analyses were performed to further investigate the transcriptional dynamics in the reconstructed *CDC25* mutants. We classified the expression levels of the *CDC25* mutants compared to that of MT8-1 by hierarchical clustering based on similarities in their expression patterns (Fig. 3a). Of the 5920 genes, the genes involved in response to stress and heat were highly upregulated in all the reconstructed *CDC25* mutants compared to the gene expression in parental MT8-1 (Cluster A). Genes in Cluster A include *HSP12*, *HSP104*, *TPS1*, and *TPS2*. Cluster B shows genes involved in membrane compositions. These upregulated genes in Cluster A and B indicate that the *CDC25* mutants have acquired thermotolerance through upregulating genes involved in stress response and organelle membrane to overcome heat stress. To identify activated transcriptional regulators in the *CDC25* mutants, we analyzed transcription factors and transcriptional activators regulating the expression of induced genes in the reconstructed *CDC25* mutants (Fig. 3b). Approximately 50–60% of the genes that were induced by more than 1.5-fold compared to that in parental MT8-1 were regulated by Msn2p and Msn4p. This result further supports the idea that the depressed activity of Cdc25p reduces intracellular cAMP levels that activates Msn2p and Msn4p through the cAMP/PKA pathway.

### Ethanol fermentation from glucose

**Figure 4:**
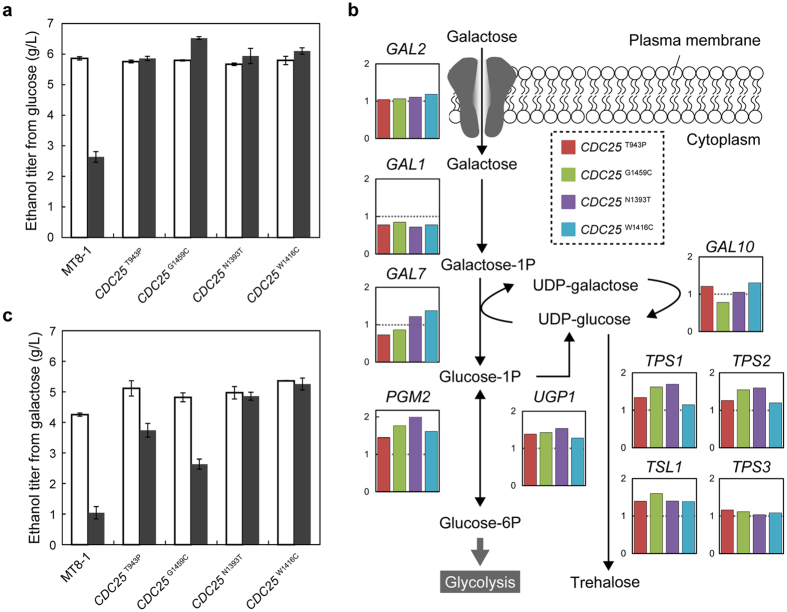
Ethanol fermentation from glucose and galactose. (**a**) Ethanol titers in 24 h from glucose at 30 °C (open bars) and 39 °C (filled bars). (**b**) Galactose fermentation pathway that partially overlaps with the trehalose synthesis pathway. The graphs represent the relative transcriptional levels of the reconstructed *CDC25* mutants compared to that of MT8-1 at 30 °C. Dashed lines indicate the transcriptional levels of MT8-1. (**c**) Ethanol titers in 24 h from galactose at 30 °C (open bars) and 39 °C (filled bars).

Next, we examined the ethanol-fermentation abilities of the *CDC25* mutants at high temperature (39 °C). Parental MT8-1 and the reconstructed *CDC25* mutants showed similar growth rates and ethanol productivities at 30 °C in glucose medium (Fig. 4a and Supplementary Fig. S1,S2). At 39 °C, however, the ethanol productivity of parental MT8-1 was greatly reduced due to heat stress. On the other hand, the reconstructed *CDC25* mutants retained ethanol productivity at the temperature. The ethanol titers of the reconstructed *CDC25* mutants reached the maximum at 24 h, while maximal ethanol titer of parental MT8-1 was observed at 36 h (Supplementary Fig. S2). The ethanol titer of reconstructed strain *CDC25*^G1459C^ in 24 h was 2.5-fold higher than that of parental MT8-1 at 39 °C. The reconstructed *CDC25* mutants had developed not only thermotolerance but also efficient ethanol fermentation ability under heat stress.

### Ethanol fermentation from galactose

In *S. cerevisiae*, galactose is metabolized through the Leloir pathway that partially overlaps with the trehalose synthesis pathway (Fig. 4b). Trehalose is a metabolite that plays an important role in thermotolerance^20^. During diauxic shift, *PGM2* and *UGP1* are induced by Msn2p and Msn4p. *PGM2* is a key enzyme for both the trehalose synthesis pathway and Leloir pathway. Therefore, we considered that the activation of Msn2p and Msn4p in the reconstructed *CDC25* mutants induced *PGM2* that would allow efficient galactose fermentation. According to transcriptome analysis performed at 30 °C, *PGM2* and *UGP1* levels were upregulated in the reconstructed *CDC25* mutants compared to that in parental MT8-1 (Fig. 4b). In addition, as expected, ethanol titers in galactose medium were slightly higher in the reconstructed *CDC25* mutants at 30 °C (Fig. 4c and Supplementary Fig. S2). We measured ethanol titers of the reconstructed *CDC25* mutants at 39 °C, because thermotolerance of the reconstructed *CDC25* mutants was considered to contribute to the ethanol productions under the heat stress. The reconstructed *CDC25* mutants, especially *CDC25*^N1393T^ and *CDC25*^W1416C^ produced more ethanol from galactose at 39 °C compared to parental MT8-1 (Fig. 4c and Supplementary Fig. S2). The ethanol titers of *CDC25*^W1416C^ in 24 h was 5.1-fold higher compared to that of MT8-1 at 39 °C. The whole-genome sequencing of intermediate strains revealed four kinds of one-point mutations in the *CDC25* gene which contributed to thermotolerance (Fig. 2a), faster growth in galactose medium (Supplementary Fig. S1), and efficient ethanol fermentation from glucose and galactose under heat stress (Fig. 4a,c and Supplementary Fig. S2).

## Discussion

In this study, 49 genomic mutations, including SNPs, insertions, and deletions were identified from stepwise bred strains (Fig. 1 and Supplementary Table S2, DDBJ accession number DRA004175). Using intermediate strains, we found *CDC25* mutations that were able to confer thermotolerance in *S. cerevisiae* (Fig. 2a). Furthermore, thermotolerant strains were successfully reconstructed with the introduction of the one-point mutations. To elucidate the underlying molecular mechanisms in the reconstructed *CDC25* mutants, the basal and induced cAMP levels were measured (Fig. 2b,c). The *CDC25* mutants accumulated less cAMP in the cells and responded poorly to glucose. Enhanced transcription of stress responsive genes was detected in all the *CDC25* mutants, indicating that the depressed cAMP/PKA pathway activated the stress responsive transcriptional activators, Msn2p and Msn4p (Fig. 3a,b). The reconstructed *CDC25* mutants maintained the ethanol fermentation rates from glucose at 39 °C and two *CDC25* mutants, *CDC25*^N1393T^ and *CDC25*^W1416C^, were able to produce ethanol from galactose at 39 °C as efficiently as at 30 °C (Fig. 4C and Supplementary Fig. S2). The ethanol titers from galactose at 39 °C were correlated to the growth rates of parental MT8-1 and the reconstructed *CDC25* mutants at 39 °C (Fig. 4c and Supplementary Fig. S1). The capabilities of *CDC25*^N1393T^ and *CDC25*^W1416C^ to grow well in galactose medium at 39 °C would lead to the efficient ethanol productions from galactose under the heat stress.

In the stepwise adaptation, we preserved and used the intermediate strains during breeding steps for the genome analysis (Fig. 1a). According to the phylogenetic tree, the 36 °C- and 38 °C-adapted populations were not derived from 32 °C- and 34 °C-adapted strains which we analyzed (Fig. 1b). The 36 °C- and 38 °C-adapted populations would have developed from a relatively minor 34 °C-adapted strain. The whole-genome analyses of intermediate strains allowed us to track individual cells and provided information about the advantageous mutations for the survival in the environment. Because reconstructed *CDC25*^N1393T^ and *CDC25*^W1416C^ grew better than *CDC25*^G1459C^ and *CDC25*^T943P^ at 39 °C (Supplementary Fig. S1), only the intermediate strains with *CDC25* (N1393T) and *CDC25* (W1416C) mutations were isolated after 34 °C-adapted populations. However, it is still unclear why only the bred mutants with the *CDC25* (W1416C) mutation were observed in the 38 °C-adapted population (Fig. 1), even though reconstructed *CDC25*^N1393T^ grew better than *CDC25*^W1416C^ at 39 °C (Supplementary Fig. S1). One possible explanation for this could be that there are additional and beneficial mutations that only a strain with the *CDC25* (W1416C) mutation had acquired in the stepwise breeding. The identification of such mutations will also facilitate the construction of thermotolerant strains.

Previous reports suggested that the cAMP-depressing mutations such as *cdc25-21* and *cdc25-22* contributed to thermotolerance in *S. cerevisiae*^21^. These *CDC25* mutations reside in the C-terminal domain that functions as GEF^22^. The N1393T, W1416C, and G1459C mutations discovered in our study are also located in the same domain. These mutations would affect the GEF activity and decrease the intracellular cAMP levels. The *cdc25-21* and *cdc25-22* mutants, however, were not able to grow as fast as the wild-type strain, because the cAMP/PKA pathway controls cell-cycle progression^9^. On the other hand, the *CDC25* mutants reconstructed in our study grew as fast as parental MT8-1 in glucose medium, and faster than parental MT8-1 in galactose medium (Supplementary Fig. S1). The different inhibitory activity of PKA against Msn2/4p and Rim15p would be able to account for thermotolerance without growth defects in the reconstructed *CDC25* mutants. A protein kinase Rim15p mainly activates transcription factor, Gis1p, which regulates cellular growth^23^. Since the activities of both Msn2/4p and Rim15p are inhibited by PKA^23^, Msn2/4p and Rim15p may competitively interact with PKA. In the reconstructed *CDC25* cells, the inhibition of only Rim15p would be preferentially maintained by the limited inhibitory activity of PKA. The substrate specificity of Tpk1p, one of the subunits of PKA, varies depending on its phosphorylation state^24^. The transcriptome analysis in this study showed that among the genes with more than 1.5-fold upregulation compared to that in parental MT8-1, only 10% was under the control of Gis1p, while approximately 50–60% was regulated by Msn2/4p (Fig. 3b). On the other hand, *cdc25-21* and *cdc25-22* might lead to the enough downregulation of PKA to inhibit both Msn2/4p and Rim15p, resulting in thermotolerance and the growth defects. The fine balance between thermotolerance and growth rates would be brought from the stepwise adaptation, because the yeast cells isolated during this adaptation process were subjected to selective pressures of both heat and growth at all times (Fig. 1a)^6^. Evolutionary engineering is one of the most effective methods of fine-tuning of signaling pathways through unpredicted mutations. *CDC25* mutants, which showed thermotolerance and similar growth rates to the parental strain, would be advantageous for fermentation, since elevated growth rates are often associated with better fermentation rate^25^.

As galactose is abundant sugars in nonfood crops as well as glucose, efficient utilization of glucose and galactose is crucial^26^. However, *S. cerevisiae* grows in galactose media at approximately half the rate than that in glucose media^27^. In *S. cerevisiae*, metabolism of galactose takes place through the Leloir pathway^28^. Metabolome analysis in a previous report indicated that the accumulation of intermediate products such as galactose-1-phosphate and glucose-1-phosphate inhibited galactose fermentation^29^. Galactose uptake and fermentation were improved by the overexpression of *PGM2*, which encodes phosphoglucomutase that mediates the conversion between glucose-1-phosphate and glucose-6-phosphate^30^. In all the reconstructed *CDC25* mutants, *PGM2* as well as other trehalose synthetic genes were induced at higher levels than those in parental MT8-1 (Fig. 4b). These genes expressions are regulated by Msn2p and Msn4p^31^. Our previous report revealed that a thermotolerant strain isolated during the stepwise adaptation induced trehalose synthesis genes and accumulated more trehalose than parental MT8-1^6^. Since the thermotolerant strain contained *CDC25* (W1416C), the reconstructed *CDC25* mutants were likely to accumulate trehalose as well as the thermotolerant strain. The downregulated cAMP/PKA pathway in the reconstructed *CDC25* mutants led to thermotolerance and efficient galactose utilization at 30 °C, and even at 39 °C.

**Figure 5:**
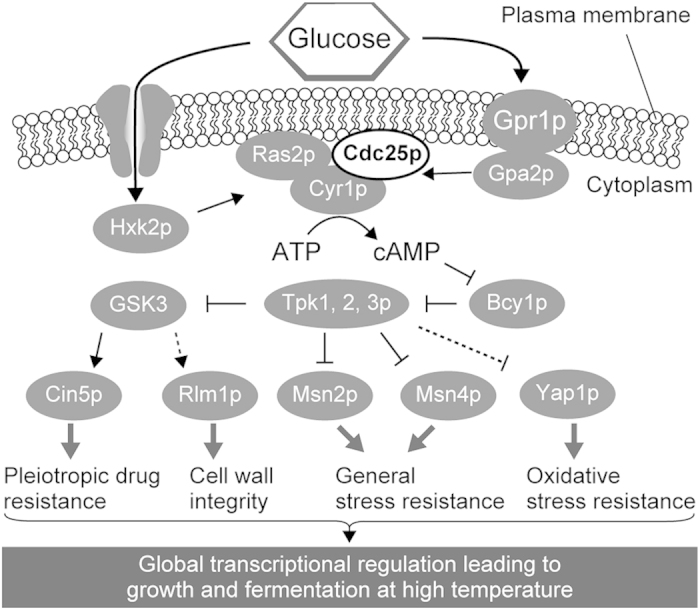
Signaling pathway in the reconstructed *CDC25* mutants. Arrows and bars represent positive and negative regulations, respectively. GSK3 indicates Rim11p, Mck1p, and Yol128c. Dashed arrows and bars represent putative or indirect regulations.

In all the four reconstructed *CDC25* mutants, the upregulated genes with more than 1.5-fold change compared to parental MT8-1, were mainly regulated by Msn2p, Msn4p, Cin5p (also called Yap4p), Rlm1p and Yap1p (Fig. 3b). This indicates that the depressed activity of Cdc25p and thus, the lower level of cAMP, activated these transcriptional regulators. Increasing intracellular level of cAMP is known to antagonize the activities of Msn2p and Msn4p through the inhibition of Bcy1p and the activation of cAMP-dependent protein kinase (PKA) composed of Tpk1p, Tpk2p, and Tpk3p (Fig. 5)^7^. The kinase is responsible for the inactivation of various transcriptional regulators including Msn2p and Msn4p^32^.

Cin5p is a transcription factor that regulates pleiotropic drug resistance and salt tolerance^33^,^34^. The phosphorylation and stability of Cin5p is positively regulated by glycogen synthase kinase 3 (GSK3) such as Rim11p, Mck1p, and Yol128c (Fig. 5)^35^. GSK3 activities are inhibited by PKA, indicating that lower intracellular cAMP levels led to the activation of Cin5p (Fig. 5).

Rlm1p is a transcription factor controlling the cell wall integrity pathway^36^. The GSK3 mutants such as *mrk1*, *mck1*, or *yol128c* showed sensitivity to SDS, zymolyase, and calcofluor white, which cause cell wall stress^37^. The mutants that lacked cAMP signaling showed thick cell walls, indicating the activation of cell wall integrity pathway^21^. The activated GSK3 might directly or indirectly activate Rlm1p (Fig. 5).

Yap1p is a transcription factor required for oxidative stress tolerance^38^. The cAMP/PKA pathway inhibits Yap1p^39^. In addition, in the absence of Bcy1p, the inhibitory subunit of PKA negatively affects the function of Yap1, suggesting that the cAMP/PKA pathway at least indirectly regulates Yap1p activity (Fig. 5)^40^.

The point mutations in *CDC25* were likely to induce multiple genes through these transcriptional regulators. In fact, *CDC25*^N1393T^, the reconstructed mutant that grew in the fastest rates at 39 °C (Supplementary Fig. S1), showed 376 genes that were expressed more than 1.5-fold higher compared to parental MT8-1. The multiple transcriptional regulators responsible for several stress tolerance mechanisms would contribute to the growth at the high temperatures, because high temperature causes not only heat stress, but also various other kinds of stresses including cell wall stress and oxidative stress (Fig. 5)^37^,^41^.

In conclusion, we found four *CDC25* mutations involved in thermotolerance and efficient glucose and galactose utilization at high temperature. Introduction of point mutations into *CDC25* conferred thermotolerance. Since Cdc25p acts upstream of the cAMP/PKA pathway, the *CDC25* mutations could comprehensively alter the transcriptional profiles through various transcriptional regulators and resulted in thermotolerance (Fig. 5). By altering the activities of transcriptional regulators, we can comprehensively alter transcriptional profiles^3^. This strategy does not need laborious genetic manipulation to organize several gene expressions; it only requires the introduction of point mutation into a gene located upstream of the signaling pathways for global transcriptional changes. The identified *CDC25* mutations will be beneficial for rational design of thermotolerant *S. cerevisiae*.

## Methods

### Yeast strains

*S. cerevisiae* MT8-1 (*MAT***a**, *ade*, *his3*, *leu2*, *trp1*, *ura3*)^42^ was used as the parental strain for breeding^6^. Thermotolerant strains were bred from MT8-1 in YPD medium (1% yeast extract, 2% peptone, 2% glucose) by stepwise adaptation to heat stress, as previously described (Fig. 1)^6^. We performed the breeding without selecting specific colonies in the adaptation steps. Strains adapted to 32 °C, 34 °C, 36 °C, and 38 °C were preserved as glycerol stocks. One-point mutations were introduced into the *CDC25* gene of MT8-1 using pAUR135 vector (Takara Bio, Shiga, Japan) following the manufacturer’s instructions. The inserts were introduced into the pAUR135 vector by the In-Fusion HD Cloning Kit (Clontech, CA, USA). The primers used for the plasmid constructions are listed in Supplementary Table S1.

### Whole-genome sequencing and RNA sequencing

Single colonies were isolated from the revived glycerol stocks. Randomly picked single colonies were separately cultivated in YPD medium for 24 h and genomic DNA was extracted by the Dr. GenTLE (from yeast) High Recovery kit (Takara Bio) and purified with AMpure XP beads (Beckman Coulter, CA, USA). Extracted genomic DNA was subjected to library construction using the Nextera DNA Sample Prep Kit (Illumina Inc., San Diego, CA, USA). For mRNA extraction, MT8-1 and *CDC25* mutants were pre-cultivated in YPD medium for 24 h. Cells were then inoculated into fresh YPD medium to be adjusted to an OD_600_ of 0.1 and cultivated at 30^o^ C for 12 h. Total RNAs were extracted with Isogen-LS (Nippon Gene, Tokyo, Japan) according to the manufacturer’s instructions. Library construction was performed using KAPA Hyper Prep Kit (Kapa Biosystems, Woburn, MA, USA). Library qualities were confirmed by an Agilent 2100 Bioanalyzer and High Sensitivity DNA Chips (Agilent Technologies, Palo Alto, CA, USA). The libraries were sequenced on Illumina MiSeq (75 bp nucleotide paired-end sequence). The average depth of each genome analysis was 30×.

### Data analysis

Paired-end reads were quality-checked with FastQC (http://www.bioinformatics.babraham.ac.uk/projects/fastqc/) and mapped to the *S. cerevisiae* genome (sacCer3) using Burrows-Wheeler Aligner^43^ for whole-genome analysis, and TopHat2^44^ for RNA-seq. To identify genomic mutations, Genome Analysis Toolkit^45^ was used. The fastq files were available at in the DDBJ data base (URL: http://trace.ddbj.nig.ac.jp/DRASearch/query? acc=dra00&show=20&sort=Study, Accession number: DRA004175). Some mutations including the *CDC25* mutations were also confirmed by DNA sequencing using ABI PRISM 310 (Thermo Fisher Scientific, Waltham, MA, USA). The phylogenetic tree was constructed based on irreversible Camin-Sokal model^46^ for all mutational events (base substitutions, insertions, and deletions). Gene expressions were estimated as fragments per kilobase of exon model per million mapped fragments (FPKM) using Cufflinks^47^. The data was utilized as the average of two independent experiments. Hierarchical clustering was performed using Cluster 3.0^48^ and visualized using Java TreeView software^49^. To identify common biological functions among genes in each cluster, DAVID^50^ was used. The *P* value threshold was set to 0.05. YEASTRACT (http://www.yeastract.com) was used to identify stress responsible transcription factors and transcriptional activators involved in gene inductions with more than 1.5-fold changes in transcriptional level compared to parental MT8-1.

### cAMP assay

Yeast cells were cultivated in the same way as for the extraction of mRNA. Cells were collected by centrifugation at 3000 × *g* for 5 min at 4 °C and washed with ice-cold water. The cell pellet was resuspended in 6% ice-cold trichloroacetic acid and 200 mg of 0.5-mm glass beads (TOMY SEIKO, Tokyo, Japan) were added. After a freeze thaw with liquid nitrogen, cells were smashed 3 times using BeadSmash 12 (WAKENYAKU, Kyoto, Japan) at 4 °C with 4000 agitations per min for 1 min. The solution was centrifuged at 2000 × *g* for 15 min at 4 °C and the supernatant was collected. HCl was added to the supernatant to a final concentration of 10 mM. The cAMP solution was extracted four times with diethyl ether and dried by lyophilization. The extracted cAMP was measured by cAMP-Screen System (Thermo Fisher Scientific). To evaluate glucose-responsiveness, cells were incubated in 0.1 mM EDTA (pH 6.0 buffered with 10 mM MES) for 2 h at 30 °C. Following the addition of glucose to a final concentration of 2%, each sample was collected at 0 min, 0.5 min, 1 min, 2 min, and 3 min, and subjected to cAMP extraction.

### Real-time PCR

Total RNAs were extracted in the same way as described above. Syntheses of cDNAs were performed using a High Capacity cDNA Transcription kit (Thermo Fisher Scientific) with 1 μg of the total RNA as a template according to the manufacture’s protocol. For the quantitative PCR, the *ACT1* gene was used as an endogenous control to normalize the expression data for each gene. The primers for real-time PCR are listed in Supplementary Table S1. Amplification was carried out using Power SYBR^®^ green PCR Master Mix (Thermo Fisher Scientific) in the 7500 Real-Time PCR System (Thermo Fisher Scientific).

### Ethanol fermentation

For ethanol fermentation, each strain was pre-cultivated in YPD medium for 24 h. Cells were inoculated into fresh YPD and YPG (1% yeast extract, 2% peptone, 2% galactose) medium to be adjusted to an optical density at 600 nm (OD_600_) of 0.5 and cultivated aerobically at 30^o^ C and 39^o^ C. Sampling was performed at every 12 h interval until 48 h. Measurement of ethanol concentration was performed as previously described^51^.

## Additional Information

**How to cite this article**: Satomura, A. *et al.* Reconstruction of thermotolerant yeast by one-point mutation identified through whole-genome analyses of adaptively-evolved strains. *Sci. Rep.* **6**, 23157; doi: 10.1038/srep23157 (2016).

## Supplementary Material

Supplementary Information

Supplementary Table
