# Supplementary material for: Reconstruction of thermotolerant yeast by one-point mutation identified through whole-genome analyses of adaptively-evolved strains
Source: Sci Rep. 2016 Mar 17;6:23157. doi: 10.1038/srep23157 (PMC4794720; doi:10.1038/srep23157)
Supplement: Supplementary Information [file srep23157-s1.pdf]

SUPPLEMENTARY INFORMATION

TITLE

Reconstruction of thermotolerant yeast by one-point mutation identified through whole-genome analyses of adaptively-evolved strains

AUTHORS AND AFFILIATIONS

Atsushi Satomura, Natsuko Miura, Kouichi Kuroda, Mitsuyoshi Ueda*

Supplementary Figure S1 Growth curves of MT8-1 and the reconstructed *CDC25* mutants in glucose and galactose media. The error bars show standard error of the mean (SEM) based on three independent measurements.

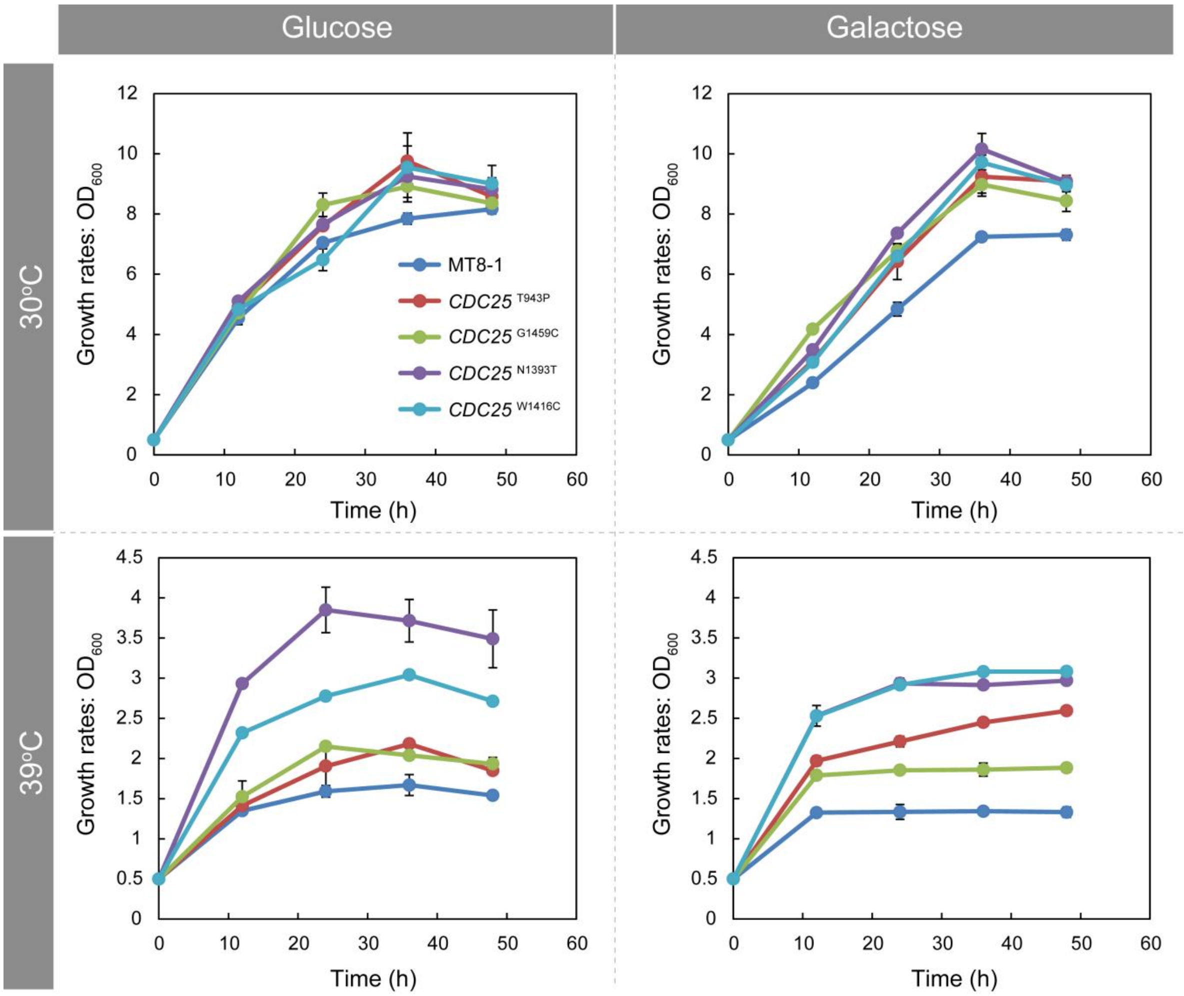

The error bars show standard error of the mean (SEM) based on three independent measurements.

Supplementary Figure S2 Ethanol productions of MT8-1 and the reconstructed *CDC25* mutants in glucose and galactose media. Ethanol was seemed to be consumed as a carbon source after 24 h at 30°C. The error bars show standard error of the mean (SEM) based on three independent measurements.

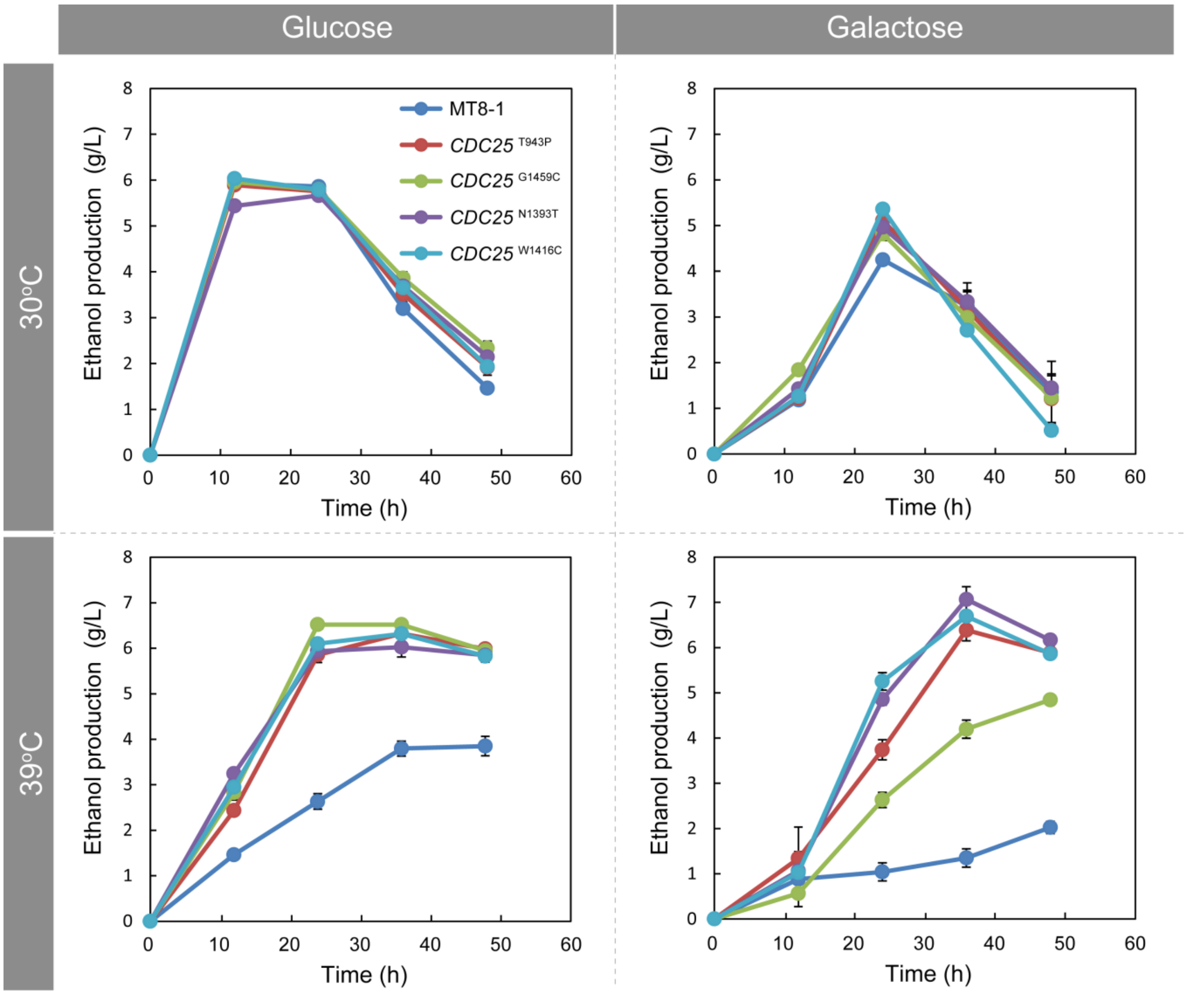

Ethanol was seemed to be consumed as a carbon source after 24 h at 30°C. The error bars show standard error of the mean (SEM) based on three independent measurements.

Supplementary Table

Table S1 Primers used in this study

Name	Sequence (5' - 3')
F *CDC25* latter	GGCTAGAGGATCCCCCTGAACTACGCAGCAAGAATGATGAAG
R *CDC25* latter	ACGACGGCCAGTGAACCTTCAGAGTTTCACTAGAGGCGC
F *CDC25* anterior	GGCTAGAGGATCCCCGGCTCACAAGATGTTCCTTAAAGAGAATC
R *CDC25* anterior	ACGACGGCCAGTGAACATATGTGTATGGGTCAATATCCAAGAGC
F *ACT1*	TCGTTCCAATTTACGCTGGTT
R *ACT1*	ACCGGCCAAATCGATTCTC
F *HSP12*	TTTGGCAGACCAAGCTAGAGATT
R *HSP12*	CGGCATCGTTCAACTTGGA
F *HSP104*	TTGAGGCCATCAAGCAACAA
R *HSP104*	AGCGCCACGAGAGTCAATTC
F *TPS1*	TACAGGTTGCAGTGCCAAGTCG
R *TPS1*	ATTGTGCGGCACCTGTGAACTC
F *TPS2*	GCAGTCCTACTGCCAACAGAAA
R *TPS2*	CTCGTTGACTTGTTGTTCCAATCT
